# Subtype Specific Differences in NS5A Domain II Reveals Involvement of Proline at Position 310 in Cyclosporine Susceptibility of Hepatitis C Virus

**DOI:** 10.3390/v4123303

**Published:** 2012-11-22

**Authors:** Israr-ul H. Ansari, Rob Striker

**Affiliations:** 1 Department of Medicine, University of Wisconsin-Madison, WI 53706, USA; Email: ihansari@wisc.edu; 2 W. S. Middleton Memorial Veteran’s Hospital, Madison, WI 53706, USA

**Keywords:** Hepatitis C virus, cyclosporine, cyclophilin, susceptibility

## Abstract

Hepatitis C virus (HCV) is susceptible to cyclosporine (CsA) and other cyclophilin (CypA) inhibitors, but the genetic basis of susceptibility is controversial. Whether genetic variation in NS5A alters cell culture susceptibility of HCV to CypA inhibition is unclear. We constructed replicons containing NS5A chimeras from genotypes 1a, 2a and 4a to test how variation in carboxy terminal regions of NS5A altered the genotype 1b CsA susceptibility. All chimeric replicons including genotype 1b Con1LN-wt replicon exhibited some cell culture sensitivity to CsA with genotype 4a being most sensitive and 1a the least. The CypA binding pattern of truncated NS5A genotypes correlated with the susceptibility of these replicons to CsA. The Con1LN-wt replicon showed increased susceptibility towards CsA when proline at position 310P was mutated to either threonine or alanine. Furthermore, a 15 amino acid long peptide fused N terminally to GFP coding sequences confirmed involvement of proline at 310 in CypA binding. Our findings are consistent with CypA acting on multiple prolines outside of the previously identified CypA binding sites. These results suggest multiple specific genetic variants between genotype 1a and 1b in the C-terminus of NS5A alter the CsA susceptibility of replicons, and some variants may oppose the effects of others.

## 1. Introduction

Nearly 200 million people are infected with HCV, and many of these people will develop severe liver disease, with or without hepatocellular carcinoma, because of persistent HCV infection. At present, HCV treatments include pegylated interferon α along with ribavirin and possibly a protease inhibitor. However, this treatment has problems with toxicity, cost, delivery, and efficacy. If severe liver disease develops and treatment fails, the only option is a liver transplant. Following liver transplant, an immunosuppressant is generally administered to help prevent graft rejection. In the majority of these cases, patients receive a calcineurin inhibitor, either cyclosporine (CsA) or tacrolimus. CsA and its nonimmunosuppressive analogs, DEBIO-25 (Alisporivir), SCY-635, and NIM811, are inhibitors of a class of cellular prolyl-peptidyl isomerases called cyclophilins (Cyps), and cyclophilin A is critical for HCV replication [1,2,3,4].

HCV has a large amount of genetic diversity and is classified in six different genotypes. The virus expresses at least 10 different proteins and several different HCV proteins have been shown to interact with cyclophilins (CypA, CypB, and possibly other cyclophilins) and modulate HCV replication [2,5,6,7,8,9]. The nonstructural protein 5A (NS5A) is a multifunctional, genetically diverse protein with three distinct hypothesized domains (DI, II and III) [10]. Whether naturally occurring variation in NS5A alters cyclophilin susceptibility has been disputed [11], but this variation could explain why an antiviral effect of cyclosporine in transplant patients may be detectable only in selected cases, if at all.

Earlier, we and others reported mutations within the DII region of NS5A which conferred resistance to CsA treatments [2,9,12,13,14]. In this study we observed varying levels of CsA susceptibility for replicons containing the carboxy terminal regions of NS5A variants of similar lengths from different HCV genotypes. The replicon containing the H77 genotype 1a sequence exhibited the least susceptibility, while the replicons containing genotype 4a and 2a chimera, as well as the genotype 1b replicon, were all more susceptible. The CsA susceptibility of these C-tail NS5A variants correlated with a CypA binding assay. Data from replicons with mutations at amino acid 310, as well as CypA binding of a peptide which contains the 310 proline, but not the defined CypA binding target PDYN, still binds CypA. Our findings are consistent with CypA acting on multiple prolines outside of the previously identified CypA binding site and suggests that conformations of NS5A adopted in viral replication can vary in cyclophilin inhibitor susceptibility. 

## 2. Results and Discussion

**Figure 1:**
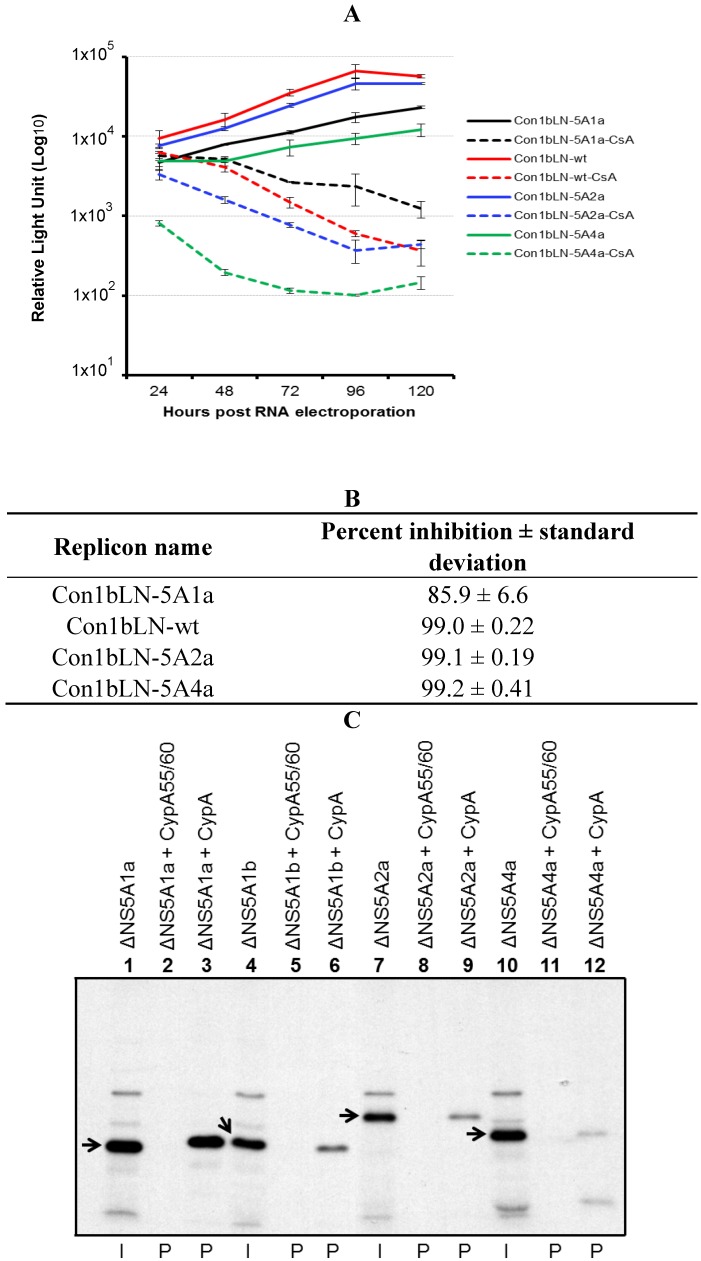
Role of HCV NS5A C-tails in CsA susceptibility and CypA binding. (**A**) The Con1bLN replicon was digested with XhoI and BstZ17I restriction enzymes (New England Biolabs) and a corresponding fragment from HCV genotype 1a genotype (aa 311–448; ARALPVWARP to TEDVVCC, accession no. AF009606) was cloned into the replicon, termed Con1bLN-5A1a. A similar strategy was used to clone genotype 2a fragment (aa 307–466; FRRPLPAWARP to EEDDTTVCC, accession no. AB047639) and genotype 4a (aa 313–449; RALPIWARPDYN to VSGSEDVVCC, accession no. Y11604.1), and termed Con1bLN-5A2a and Con1bLN-5A4a. The Huh7.5 cells were electroporated with *in vitro* synthesized RNA derived from Con1bLN-5A1a, Con1bLN-5A2a, Con1bLN-5A4a and Con1bLN-wt replicons. Equal numbers of electroporated cells were plated. The cells were either untreated (solid lines) or treated with CsA (dotted lines) for 120 hrs and luciferase activity was monitored every 24 hrs and presented. (**B**) The percent inhibition of respective replicons in (A) were calculated and presented. (**C**) The CypA binding capacity of NS5A regions derived from different genotypes. The ^35^S labeled proteins were incubated with either GST-CypA or GST-CypA55/60. The pulled down complexes were resolved by SDS-PAGE and signal was detected after autoradiography. The arrows indicate expected size of polyprotein (I = input (1/20th loaded); P = pull-down).

Several HCV subgenomic replicons have been described. However, replicons vary in the robustness of replication, and obviously vary genetically at several different places in the genome. To avoid the lower replication capacity associated with certain HCV genotypes and isolate the NS5A variation between genotypes, we constructed NS5A chimeric replicons in the backbone of the commonly used genotype 1b Con1 replicon which displays robust replication capacity. The *in vitro* transcribed RNAs derived from the Con1bLN-wt (wild type), and chimeric replicons containing other NS5A genotypic sequences from amino acid 312 to the NS5A-NS5B cleavage site (Con1bLN-5A1a, Con1bLN-5A2a and Con1bLN-5A4a), were electroporated into Huh7.5 cells and luciferase activity was monitored over a period of five days in the presence and absence of CsA. As shown in Figure 1A, all the replicons exhibited similar replication kinetics in the absence of CsA, thus indicating that the replaced polypeptide derived from genotypes 1a, 2a and 4a did not have deleterious effects on viral replication (red, blue, black and green lines). However the same replicons displayed contrasting susceptibility upon CsA treatment. The Con1bLN-5A4a replicon was found to be most susceptible (solid green *vs.* dotted green lines, almost 100-fold less replication, Figure 1B) to CsA treatment among all replicons. Although the Con1bLN-5A1a replicon had slightly lower replication capacity than the Con1bLN-wt replicon, the Con1bLN-5A1a replicon displayed the least susceptibility to CsA treatment (solid black *vs.* dotted black lines, only 10-fold less replication compared to no CsA treatment). The Con1LN-wt and Con1LN-5A2a replicons had slightly better replication capacity than the Con1LN-5A1a and Con1LN-5A4a replicons in the absence of CsA, and showed less inhibition to CsA treatment compared to Con1LN-5A1a replicon (red and blue lines). 

By making NS5A chimeras, we directly compared the cyclosporine susceptibility of specific NS5A sequences without the confounding effects of other parts of the genome. Due to the diversity of each subtype, our results do not imply that every genotype 1a HCV is less susceptible than every genotype 1b, only that there is a difference between H77 1a and Con1 in this carboxy terminal region of NS5A. Previous studies indicate the NS5A derived from different genotypes and the strong conservation of the PDYN binding site for CypA identified by NMR has been used to argue that cyclophilin inhibitors are “pangenotypic” and the heterogeneity of NS5A does not correlate with cyclophilin inhibition [11]. Our data suggests most, if not all, HCV are susceptible to cyclophilin inhibitors. To our knowledge, this is the first demonstration that genotype 4 HCV is likely CsA susceptible, but our data suggests that NS5A polymorphisms outside the conserved PDYN sequence previously associated with CypA binding can also influence the degree of CsA susceptibility. 

To test the interaction between NS5A genotypic carboxy terminal regions and CypA, we expressed NS5A polypeptides derived from different genotypes in a cell-free translation system in the presence of ^35^S cysteine/methionine and performed the CypA binding assay as previously described [12]. We observed the polypeptide derived from genotype 1a bound more efficiently than the corresponding polypeptides of 1b, 2a and 4a genotypes (Figure 1C). The polypeptides derived from genotype 1b and 2a bound to CypA but with less efficiency compared to genotype 1a (input lanes; 4 and 7, pull-down lanes; 6 and 9). The apparent migration of protein derived from 2a genotype of similar length in SDS-PAGE was slower compared to 1b genotype due to the presence of an additional 23 amino acids. The genotype 4a polypeptide displayed the least CypA binding in similar experimental set up (input lane 10 with pull-down lane 12). In general, these CypA binding patterns correlated well with their respective replicons’ susceptibility towards CsA (Figure 1A). In all the pull-down assays, an active site mutant protein CypA55/60 was used as negative control. Overall, the CypA binding data indicates that the carboxy terminal regions of NS5A both interact with CypA as expected and correlate to some degree with the ability of the replicon to replicate in the presence of CsA. 

**Figure 2:**
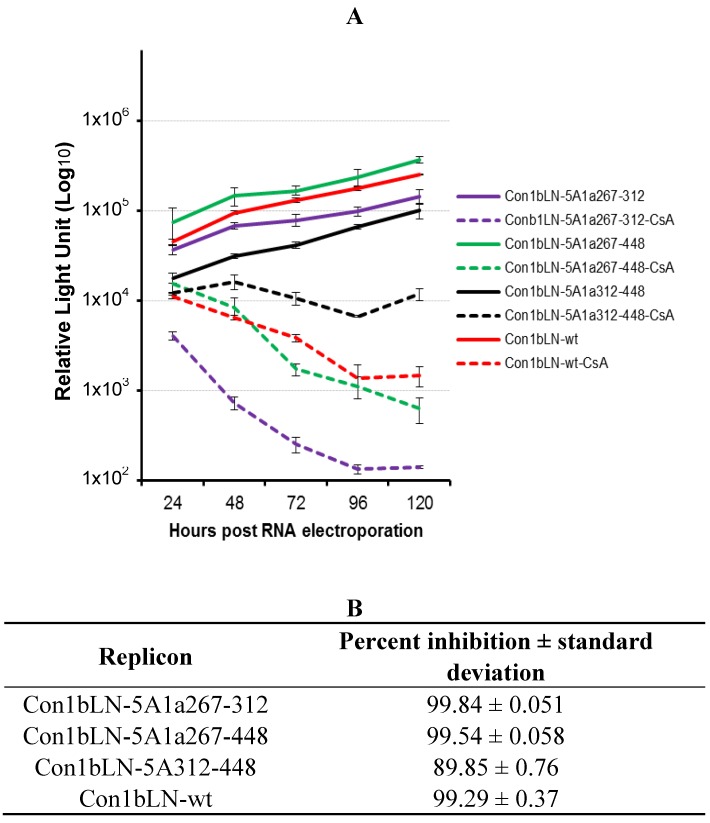
Con1LN-wt replicons containing different lengths of the carboxy terminus of NS5A genotype 1a have different susceptibility to CsA. (**A**) The CsA susceptibility replication assay of Con1bLN-5A1a-267-312, Con1bLN-5A1a-267-448 and Con1bLN-5A1a-312-448 (referring to same construct in Figure 1A; Con1LN-5A1a) replicons was performed as described above. (**B**) The percent inhibition of respective replicons in (A) were calculated and presented.

**Figure 3:**
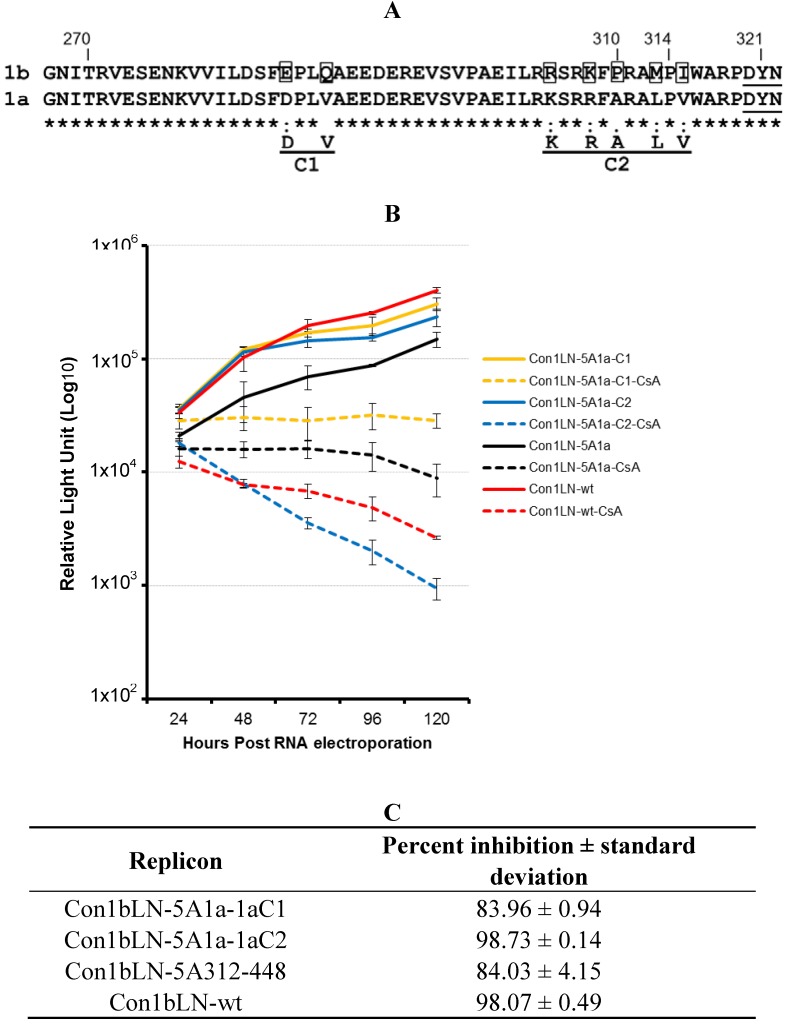
Mutational analysis of Con1bLN-5A1a chimericreplicons reveal linear NS5A regions that alter CsA susceptibility. (**A**) The HCV 1b (358 isolates) and 1a (224 isolates) amino acid sequences were retrieved from the European HCV Database and subjected to amino acid homology analysis using web based program . The amino acid alignment analysis was performed on 53 amino acids N-terminal to the DYN region of HCV NS5A from 1b and 1a genotypes. The highly conserved DYN region is underlined. Site-directed mutagenesis was performed in two cluster regions, C1 and C2, in Con1bLN-wt replicon to make it similar to genotype 1a. The boxed residues were substituted in 1b replicon with genotype 1a amino acids. Amino acids of interest are numbered. The amino acids of interest are numbered. (**B**) The CsA susceptibility replication assay was performed on replicons Con1bLN-1aC1, Con1bLN-1aC2, Con1bLN-5A1a and Con1bLN-wt replicons. (**C**) The percent inhibition of respective replicons in (B) were calculated and presented.

**Figure 4:**
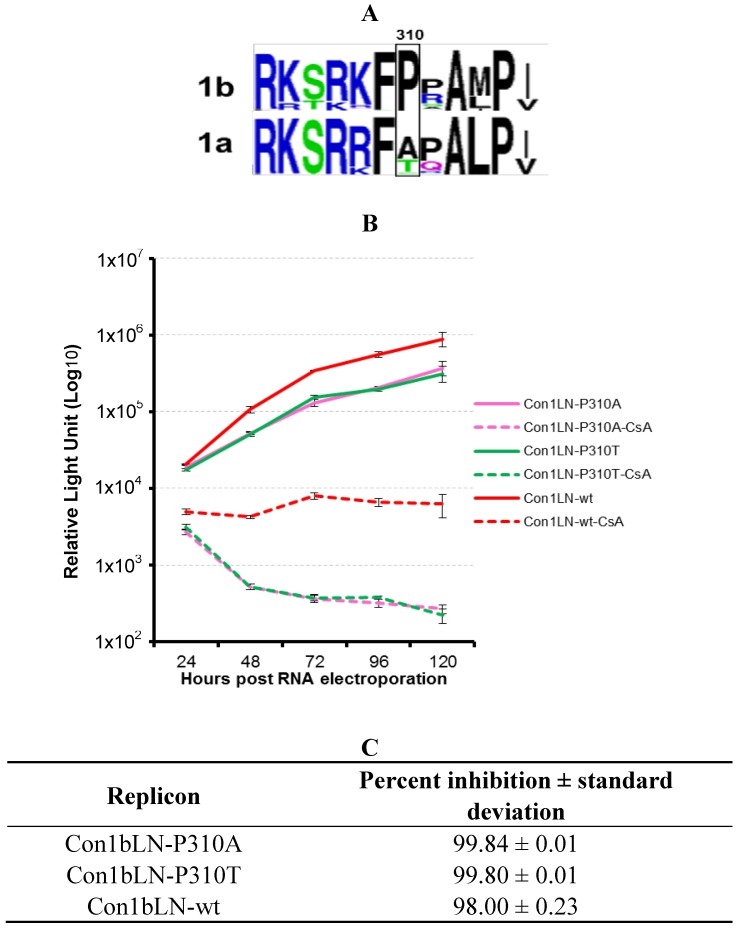
Mutational analysis of Con1bLN-wt replicon at position 310 and analysis of CsA susceptibility. (**A**) Logo analysis of the C2 region of 358 isolates from genotype 1b and 224 from genotype 1a of HCV NS5A. The boxed proline residue at position 310 appears to be highly conserved among most HCV 1b viruses. Sequences derived from genotype 1b and 1a are marked. (**B**) The CsA susceptibility of Con1bLN-P310A, Con1bLN-P310T was compared to Con1bLN-wt replicons. (**C**) The percent inhibition of respective replicons in (B) were calculated and presented.

We noticed a limited number of differences in the H77 1a genotype compared to the Con1b in the region, approximately 50 amino acids N terminal to WARPDYN. We therefore decided to explore the role of this region towards CsA susceptibility. We constructed two additional chimeric replicons encompassing amino acids 267–312, and the 267–448 region derived from genotype 1a in the backbone of Con1LN-wt replicon, and compared their susceptibility to that of Con1bLN-5A1a (here after known as Con1bLN-5A1a312-448). These replicons replicated well in the absence of CsA but had different degrees of susceptibility to CsA (Figure 2A). The replicon containing the 1a region from 267–312 (purple lines) was the most sensitive to CsA (~3 log, ~99% reduction, Figure 2B) suggesting that the 267–312 1a stretch conferred additional susceptibility to Con1bLN-wt replicon. While the Con1bLN-wt (dotted red) and the Con1bLN-5A267-448 replicon (dotted green lines) displayed similar CsA-like susceptibility to the Con1bLN-5A1a267-312 at 96 hours in Figure 2B, notice the clear separation at an earlier time point in Figure 2A (compare dotted purple to dotted red/green). This suggested to us the relatively increased susceptibility of Con1bLN-5A1a267-448 replicon, as compared to Con1bLN-5A1a312-448, which was due to the loss of the 267–3121b region. Since there were fewer consistent differences between 1b and 1a amino terminal to 312 compared to the carboxy terminal, we attempted to isolate a subtype “1a susceptibility” feature amino terminal to 312, rather than the “1a relative resistance” feature demonstrated in Figure 1. Therefore, an effort was made to define the particular residue that alters CsA susceptibility in cell culture in the context of the replicon Con1bLN-5A1a312-448 that exhibits resistance. Shown in Figure 3A is the amino acids sequence homology between genotype 1a and 1b amino acids N-terminal to an extremely high conserved region, WARPDYN, but within the NS5A region that contributes most to CypA binding as observed in our previous findings. We observed two distinct clusters that have noticeable amino acids changes, named Cluster1 (C1) and Cluster2 (C2) (Figure 3A). These mutations were incorporated into Con1bLN-5A1a312-448 chimeric replicon termed Con1bLN-1aC1 and Con1bLN-1aC2 such that the genotype 1a residues at C1 and C2 replaced the residues present in genotype 1b. The Con1bLN-5A-1aC1 replicon (yellow lines) was competent enough for replication in both the presence and absence of CsA, thereby indicating that mutating amino acids EQ to DV did not affect genome replication following CsA treatment (Figure 3B) and maintained similar CsA susceptibility compare to Con1bLN-5A1a312-448. On the other hand, when a 1a stretch of C2 was present in the Con1bLN-1aC2 replicon, there was increased susceptibility in the presence of CsA and produced ~3 log reduction, suggesting that a single or a combination of amino acids within C2 (RKPMI) are capable of altering CsA susceptibility. To our knowledge, this is the first observation that variation ~10 amino acids N-terminal to WARPDYN in genotype 1 alters cyclosporine susceptibility and is consistent with multiple prolines in this region being influenced by cyclophilin, as shown before [13,15]. Only mutations in the C2 cluster resulted in increased sensitivity to CsA, but not in cluster 1. Further analysis of amino acid residues present in C2 among genotype 1a reveals this stretch (RKSRRF**A**RALPV) of amino acids are fairly conserved, except the alanine (Figure 3A). The H77 1a genotype (AF009606) has alanine (bold underlined) at this particular position, compared to a proline in 1b. Logo analysis [16] of amino acids in C2 cluster of 1a and 1b sequences demonstrates that while proline is well conserved in the genotype 1b lineage, genotype 1a commonly has either an alanine or a threonine (Figure 4A). 

**Figure 5:**
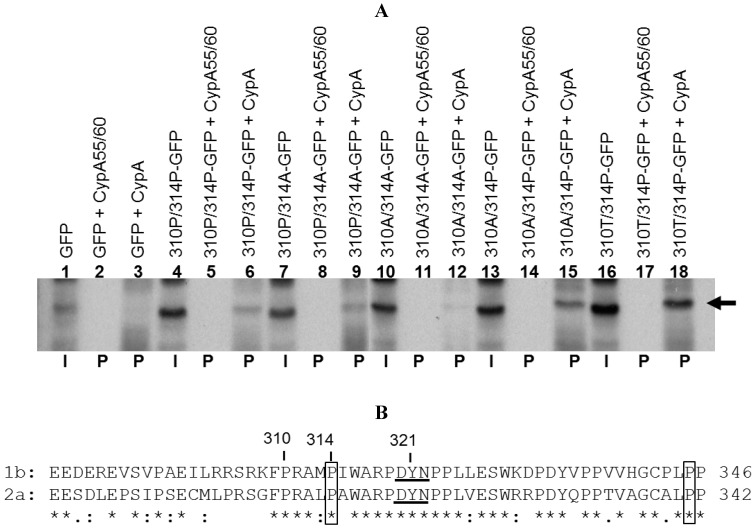
CypA binding analysis of a peptide containing Ala, Pro and Thr at position 310. (**A**) ^35^S labeled proteins derived from NS5A peptide fused to the GFP coding sequences were incubated with either GST-CypA55/60 or with GST-CypA. The pulled-down complexes were resolved by SDS-PAGE and signal was detected by autoradiography. The arrow indicates expected size proteins (I = input (1/20th loaded); P = pull-down). (**B**) Alignment CypA binding region from genotype 1b and 2a. The highly conserved DYN region is underlined and amino acids of interests are numbered. In addition to P310 described in this study, the boxed residues were demonstrated previously to regulate the Alisporivir susceptibility in genotype 2a [13].

To examine the role of amino acid 310 in genotype 1b (Figure 4A) in its native genotype 1b context, we mutated this amino acid to either an alanine or threonine. The resulting replicons Con1bLN-P310A and Con1bLN-P310T were tested for CsA susceptibility as described above. Both the replicons carrying mutations Ala or Thr became more sensitive to CsA treatment than the Con1bLN-wt replicon indicating the role of proline at position 310 in CypA regulation in genotype 1b (Figure 4B). We next tested if CypA could bind to this stretch of amino acids and, if so, whether or not a proline at 310 and/or 314 altered binding. A 15 amino acid long peptide representing this region was engineered as an N-terminal fusion protein with GFP and GST-CypA binding assay was performed as above. The GFP alone did not bind to either CypA55/60 or GST-CypA in a pull-down assay (Figure 5A, lane 2 and 3). The peptide tagged GFP carrying 310P/314P amino acids bound well (lane 6), whereas a peptide tagged GFP containing 310A/314A amino acids exhibited little to no binding to CypA (lane 12), thus indicating that one or both of the prolines may contribute to CypA binding. We then expressed GFP tagged with peptides carrying mutations at the 310 and 314 positions to confirm which prolines contribute to CsA susceptibility. The peptide tagged GFP carrying mutation at 310P/314A (lane 9) and 310A/314P (lane 15) bound to CypA equally well as 310P/314P, thereby indicating that both the prolines contribute to CypA binding. Furthermore, mutating proline at position 310 to Ala or Thr did not abolish the CypA binding completely (lanes 15 and 18) partly due to the fact that both peptides 310A/314P and 310T/314P contain proline at 314 positions. However, a single point mutation at position 310 to either Ala or Thr rendered the 1b replicon more sensitive to CsA, indicating the proline at 310 has a role in CypA binding. Although we and others [13] observed a contribution of 314P to CypA binding, our data indicates that 310 can also participate in CypA binding and contributes significantly directly or indirectly towards CsA susceptibility in tissue culture. The amino acid analysis of the C2 region also indicates that proline (P310) is highly conserved in non‑genotype 1a HCV. Interestingly, this proline residue, along with two other prolines (P310 and P315) in genotype 2a, have been found to be in the direct vicinity of NS5A:CypA interaction region when tested by gel filtration, circular dichroism, and NMR spectroscopy [15]. Our transient replication data demonstrate mutating residues in the C2 region, and, in particular, genotype 1b P310 to alanine or threonine residue led to increased susceptibility of 1b replicon (pink and green lines), thus indicating the region’s critical involvement in CsA susceptibility. Our data is consistent with the genotype 2a data on P310 (homologous to P314 in genotype 1b), as well as P342 of genotype 2a in CsA regulation [13], indicating critical roles of amino acids N-terminal as well as C-terminal to the DYN motif. The role of residue P306 in genotype 2a (corresponding to 310 in genotype 1b identified in this study) in the cyclophilin inhibitor Alisporivir susceptibility to our knowledge has not been investigated (Figure 5B). 

## 3. Experimental Section

The short stretch of carboxy terminal regions of different genotypic variants of HCV NS5A were PCR amplified and cloned directionally in the Con1b-LN replicon . The forward and reverse primers to amplify genotypes 1a and 4a were designed to contain the XhoI and BstZ17I restrictions enzymes respectively, whereas primers to amplify genotypes 2a contained SalI and BstZ17I restrictions enzymes. The plasmid DNA was linearized with XbaI and used for *in vitro* RNA synthesis using MEGAscript T7 kit (Invitrogen). To test the CsA susceptibility of Con1bLN-wt and chimeric replicons, the *in vitro* synthesized RNA was electroporated in Huh7.5 cells and equal number of cells were plated in 24-well plates. The electroporated cells were either treated with CsA 0.5 µg/mL CsA or left untreated. The cells were lysed with 100 µL of renilla luciferase lysis buffer, and 5 µL of cleared lysate was used to evaluate luciferase activity using the Renilla Luciferase Assay system (Promega) [12,17]. Additional mutations were generated in either Con1bLN-wt or chimeric replicons by a standard primer-driven PCR. All the clones were sequence verified to confirm the desired mutations.

The PCR fragments corresponding to the 311–447 region in genotypes 1a, 1b, 2a and 4a of NS5A was PCR amplified and were cloned in T7-based expression vector, termed as ΔNS5A1a, ΔNS5A1b, ΔNS5A2a and ΔNS5A4a. All the clones were sequence verified before protein expression. The cell‑free translation was performed using TnT T7 Quick Coupled Transcription/Translation System (Promega, Madison, WI, USA) in the presence of EasyTag™ EXPRESS35S Protein Labeling Mix, [^35^S] (Perkin Elmer) and subsequent CypA binding was performed as described previously [12]. In brief, the ^35^S labeled polypeptides from different NS5A carboxy termini were incubated with either GST‑CypA55/60 or with GST-CypA overnight at 4 °C. The GST-CypA55/60 is an active site mutant version GST‑CypA where amino acids R55 and F60 are mutated to alanine, respectively. The bound complexes were washed five times with PBS containing 0.25% NP-40 with shaking every 5 minutes at 4 °C. The complexes were resolved by SDS-12%PAGE and exposed to an X-ray film. A similar CypA binding strategy was performed for peptide tagged GFP expressed proteins. Briefly, a 15 amino acid long peptide (LRRSRKF**P**RAM**P**IWA) was genetically engineered to be fused N terminally to the GFP coding sequence. The protein was expressed as above and was used for CypA-binding analysis as described above. All the constructs described above were sequenced to confirm desired mutations. 

## 4. Conclusions

Here, we have shown specific differences in cyclophilin inhibitor susceptibility between the NS5A of genotype 1a and genotype 1b, as well as the susceptibility of genotype 4. All the replicons displayed similar kinetics of replication in the absence of CsA, indicating the substituted regions of NS5A did not alter general replication. While an undetermined sequence carboxy terminal to 312 in genotype 1a exhibited more CsA resistance in Figure 1, we were able to identify 1a residues at position 310, which conferred some degree of increased susceptibility relative to genotype 1b. Furthermore, the CypA binding pattern of these peptides was in agreement with genome replication; *i.e.*, better CypA binding correlated with better replication in the presence of CsA. A limitation of our study is that chimeric replicons may not behave identically to the sequence in the proper subtype context. Mutational analysis amino terminal to the well-accepted PDYN Cyp binding site [2,9,12,14,15] confirmed the involvement of a proline residue present at position 310 in genotype 1b in determining the level of CsA susceptibility. These subtle variations between genotypes may make developing NS5A sequence-based rules to predict the degree of cyclophilin inhibitor susceptibility challenging. Non‑immunosuppressive cyclophilin inhibitors, including Alisporivir, are in late-stage clinical trials and have demonstrated efficacy including a genotype 3 patient being cured by a short duration alisporivir monotherapy [3,18]. While the approval of protease inhibitors has greatly increased the possibility of curing HCV, small molecule inhibitors quickly select resistance in HCV unless given in combination with other antivirals with different mechanisms of action.

## References

[1] Vermehren J., Sarrazin C. (2011). New HCV therapies on the horizon. Clin. Microbiol. Infect..

[2] Fernandes F., Poole D.S., Hoover S., Middleton R., Andrei A.C., Gerstner J., Striker R. (2007). Sensitivity of hepatitis C virus to cyclosporine A depends on nonstructural proteins NS5A and NS5B. Hepatology.

[3] Flisiak R., Horban A., Gallay P., Bobardt M., Selvarajah S., Wiercinska-Drapalo A., Siwak E., Cielniak I., Higersberger J., Kierkus J. (2008). The cyclophilin inhibitor Debio-025 shows potent anti-hepatitis C effect in patients coinfected with hepatitis C and human immunodeficiency virus. Hepatology.

[4] Ma S., Boerner J.E., TiongYip C., Weidmann B., Ryder N.S., Cooreman M.P., Lin K. (2006). NIM811, a cyclophilin inhibitor, exhibits potent *in vitro* activity against hepatitis C virus alone or in combination with alpha interferon. Antimicrob. Agents Chemother..

[5] Watashi K., Ishii N., Hijikata M., Inoue D., Murata T., Miyanari Y., Shimotohno K. (2005). Cyclophilin B is a functional regulator of hepatitis C virus RNA polymerase. Mol. Cell.

[6] Yang F., Robotham J.M., Nelson H.B., Irsigler A., Kenworthy R., Tang H. (2008). Cyclophilin A is an essential cofactor for hepatitis C virus infection and the principal mediator of cyclosporine resistance *in vitro*. J. Virol..

[7] Foster T.L., Gallay P., Stonehouse N.J., Harris M. (2011). Cyclophilin A interacts with domain II of hepatitis C virus NS5A and stimulates RNA binding in an isomerase-dependent manner. J. Virol..

[8] Ciesek S., Steinmann E., Wedemeyer H., Manns M.P., Neyts J., Tautz N., Madan V., Bartenschlager R., von Hahn T., Pietschmann T. (2009). Cyclosporine A inhibits hepatitis C virus nonstructural protein 2 through cyclophilin A. Hepatology.

[9] Yang F., Robotham J.M., Grise H., Frausto S., Madan V., Zayas M., Bartenschlager R., Robinson M., Greenstein3 A.E., Nag A. (2010). A major determinant of cyclophilin dependence and cyclosporine susceptibility of hepatitis C virus identified by a genetic approach. PLoS Pathog..

[10] Tellinghuisen T.L., Foss K.L., Treadaway J.C., Rice C.M. (2008). Identification of residues required for RNA replication in domains II and III of the hepatitis C virus NS5A protein. J. Virol..

[11] Chatterji U., Lim P., Bobardt M.D., Wieland S., Cordek D.G., Vuagniaux G., Chisari F., Cameron C.E., Targett-Adams P., Parkinson T. (2010). HCV resistance to cyclosporin A does not correlate with a resistance of the NS5A-cyclophilin A interaction to cyclophilin inhibitors. J. Hepatol..

[12] Fernandes F., Ansari I.U., Striker R. (2010). cyclosporine inhibits a direct interaction between cyclophilins and hepatitis C NS5A. PLoS One.

[13] Grise H., Frausto S., Logan T., Tang H. (2012). A conserved tandem cyclophilin-binding site in hepatitis C virus nonstructural protein 5A regulates alisporivir susceptibility. J. Virol..

[14] Puyang X., Poulin D.L., Mathy J.E., Anderson L.J., Ma S., Fang Z., Zhu S., Lin K., Fujimoto R., Compton T. (2010). Mechanism of resistance of hepatitis C virus replicons to structurally distinct cyclophilin inhibitors. Antimicrob. Agents Chemother..

[15] Hanoulle X., Badillo A., Wieruszeski J.M., Verdegem D., Landrieu I., Bartenschlager R., Penin F., Lippens G. (2009). Hepatitis C virus NS5A protein is a substrate for the peptidyl-prolylcis/trans isomerase activity of cyclophilins A and B. J. Biol. Chem..

[16] Crooks G.E., Hon G., Chandonia J.M., Brenner S.E. (2004). WebLogo: a sequence logo generator. Genome. Res..

[17] Lohmann V., Korner F., Koch J., Herian U., Theilmann L., Bartenschlager R. (1999). Replication of subgenomic hepatitis C virus RNAs in a hepatoma cell line. Science.

[18] Patel H., Heathcote E.J. (2011). Sustained virological response with 29 days of Debio 025 monotherapy in hepatitis C virus genotype 3. Gut.

